# Population structure of sea-type and lake-type sockeye salmon and kokanee in the Fraser River and Columbia River drainages

**DOI:** 10.1371/journal.pone.0183713

**Published:** 2017-09-08

**Authors:** Terry D. Beacham, Ruth E. Withler

**Affiliations:** Fisheries and Oceans Canada, Pacific Biological Station, Nanaimo, B. C. Canada; National Cheng Kung University, TAIWAN

## Abstract

Population structure of three ecotypes of *Oncorhynchus nerka* (sea-type Sockeye Salmon, lake-type Sockeye Salmon, and Kokanee) in the Fraser River and Columbia River drainages was examined with microsatellite variation, with the main focus as to whether Kokanee population structure within the Fraser River drainage suggested either a monophyletic or polyphyletic origin of the ecotype within the drainage. Variation at 14 microsatellite loci was surveyed for sea-type and lake-type Sockeye Salmon and Kokanee sampled from 121 populations in the two river drainages. An index of genetic differentiation, F_ST_, over all populations and loci was 0.087, with individual locus values ranging from 0.031 to 0.172. Standardized to an ecotype sample size of 275 individuals, the least genetically diverse ecotype was sea-type Sockeye Salmon with 203 alleles, whereas Kokanee displayed the greatest number of alleles (260 alleles), with lake-type Sockeye Salmon intermediate (241 alleles). Kokanee populations from the Columbia River drainage (Okanagan Lake, Kootenay Lake), the South Thompson River (a major Fraser River tributary) drainage populations, and the mid-Fraser River populations all clustered together in a neighbor-joining analysis, indicative of a monophyletic origin of the Kokanee ecotype in these regions, likely reflecting the origin of salmon radiating from a refuge after the last glaciation period. However, upstream of the mid-Fraser River populations, there were closer relationships between the lake-type Sockeye Salmon ecotype and the Kokanee ecotype, indicative of the Kokanee ecotype evolving independently from the lake-type Sockeye Salmon ecotype in parallel radiation. Kokanee population structure within the entire Fraser River drainage suggested a polyphyletic origin of the ecotype within the drainage. Studies employing geographically restricted population sampling may not outline accurately the phylogenetic history of salmonid ecotypes.

## Introduction

The Pacific salmon species Sockeye Salmon *Oncorhynchus nerka* is characterized by three main ecotypes that are distinguished by differences in life history in fresh water. The “lake-type” ecotype typically spawns in lakes, or in tributaries associated with lakes, their offspring rear in these nursery lakes for at least one year before migrating to the ocean [1], and it is generally the most widespread and abundant life history type. However, where lake-rearing habitat is inaccessible or unavailable, the second ecotype can be common, where Sockeye Salmon spawn in tributaries or mainstem side channels, and the juveniles rear for several months in estuarine waters (“sea-type”) or at least one year (“river-type”) in the river environment before migrating to the ocean [2, 3]. Sea-type and river-type Sockeye Salmon are similar in that the juveniles both rear in river habitats prior to smolt migration to the ocean, but sea-type juveniles do not spend a winter in fresh water, and thus lack a freshwater annulus. The river-type form has been considered to be a special case of the sea-type form because neither type rear in lakes [4]. At maturity, both ecotypes undertake an anadromous migration, returning from the ocean to spawn in fresh water in their natal rivers. The third ecotype is commonly known as Kokanee, in which individuals in this ecotype are non-anadromous and complete their life cycle entirely in fresh water [5]. Within each ecotype, there can be differentiation with respect to spawning locations, with adults spawning on beaches within lakes, or in tributary rivers and streams [6, 7, 8, 9]. There is also evidence to suggest that in salmonids alternative migratory tactics co-exist within populations, and that all individuals may potentially adopt any of the alternative phenotypes or ecotypes [10].

The evolutionary relationships among the lake-type, sea-type, and Kokanee ecotypes have been a matter of continuing interest. The components of a “recurrent evolution” hypothesis for *Oncorhynchus nerka* have been outlined previously [4]. The basic components of the hypothesis included the following three main assumptions. The sea-type ecotype was considered a genetically-diverse ancestral form with poorly genetically differentiated populations, and straying by this ecotype allowed new habitats to be colonized after glacial retreat. Once the lake habitat became accessible and productive, genetically differentiated lake-type Sockeye Salmon evolved repeatedly from the sea-type ecotype in parallel adaptive radiations. When the lake environment became sufficiently productive, then the fitness of nonanadromous individuals was postulated to become at least equivalent to that of anadromous individuals, and consequently populations of the Kokanee ecotype evolved independently from the lake-type Sockeye Salmon ecotype in a parallel adaptive radiation.

An alternative perspective on Kokanee evolution was provided by [11], with allozyme frequencies for Kokanee populations from a portion of the Fraser River and Columbia River drainages more similar to each other than either was to allozyme frequencies for their respective sympatric Sockeye Salmon populations. In this perspective, it is assumed that Kokanee populations in the Fraser River and Columbia may share a common monophyletic origin relative to their sympatric Sockeye Salmon counterparts, and that present day population structure of Kokanee reflects radiation from a glacial refugium and gene exchange between these two river basins, rather than independent parallel evolution from the lake-type Sockeye Salmon ecotype within each basin. The key question to evaluate in the current study was whether the Kokanee ecotype in the Fraser River and Columbia River drainages evolved independently in parallel adaptive radiation from the lake-type Sockeye Salmon ecotype [4] or whether the Kokanee ecotype in both river drainages share a common monophyletic origin [11]. Earlier studies using allozymes showed distinct differences between the sea-type and lake-type ecotype population structure. Differentiation among lake-type populations was attributed to strong homing fidelity to their natal streams, whereas a lack of differentiation among sea-type populations was interpreted as reflecting high rates of straying among populations [3, 12]. Later studies with microsatellites on the population structure of the lake-type and sea-type ecotypes indicated that a regional structuring of populations was observed, with populations typically clustered within lakes and river drainages [13]. Within British Columbia, there was evidence of genetic differentiation among sea-type populations inhabiting different river drainages, with those in the Alsek River distinct from those in the Stikine and Taku rivers in northern British Columbia, and with those in the Nass River and Fraser River distinct from those in the Alsek, Taku, and Stikine river drainages [14].

In the current study, we outline the results of a survey of microsatellite variation of the sea-type Sockeye Salmon, lake-type Sockeye Salmon, and Kokanee ecotypes of *O*. *nerka* populations in the Fraser River and Columbia River drainages, and evaluate the recurrent evolution hypothesis outlined by [4] with reference to the three main assumptions of *O*. *nerka* evolution. Comparisons are conducted between the level of genetic diversity observed for two sea-type populations in the lower Fraser River drainage relative to that observed between Sockeye Salmon and Kokanee populations within the Fraser River and Columbia River drainages, with the analysis conducted by comparisons of heterozygosity as well as comparing the number of alleles observed in each ecotype standardized to a common sample size. Next, we evaluate whether genetically differentiated lake-type Sockeye Salmon have evolved repeatedly from the sea-type ecotype in parallel adaptive radiations. If so, then genetic differences between sea-type and lake-type ecotypes within a geographic region should be less than differences among regions within the lake-type ecotype, with the analysis conducted by comparisons of genetic differentiation (*F*_*ST*_) among ecotypes and regions. Finally, we evaluate whether the Kokanee ecotype evolved independently from the lake-type Sockeye Salmon ecotype by comparing the level of differentiation within and between ecotypes from the same geographic region. If Kokanee populations in the Fraser River and Columbia River drainages share a common monophyletic origin, then differentiation between the ecotypes should be greater than differentiation among regions within an ecotype, with the evaluation conducted through gene diversity and cluster analysis.

## Results

### Variation within populations

Variation was observed in the number of alleles at the 14 microsatellite loci surveyed in the study. The fewest number of alleles was observed at *Oki1a* (4 alleles sea-type Sockeye Salmon ecotype, 8 alleles lake-type Sockeye Salmon and Kokanee ecotypes), and the greatest number of alleles was observed at *Oki29* (23 alleles sea-type Sockeye Salmon ecotype, 36 alleles lake-type Sockeye Salmon ecotype, 37 alleles Kokanee ecotype). The number of alleles observed displayed considerable variation among the three ecotypes of *O*. *nerka*. After standardization to a common sample size, the sea-type Sockeye Salmon ecotype displayed considerably fewer alleles (203 alleles, P<0.05) across the 14 microsatellite loci than did either the lake-type Sockeye Salmon (241 alleles) or Kokanee (260 alleles) ecotypes (Table 1). The largest differences in number of alleles between sea-type Sockeye Salmon and Kokanee ecotypes were observed at *Oki16* (14 alleles), *Ots100* (8 alleles), *Oki6* (6 alleles), and *Oki29* (6 alleles). Average expected heterozygosity in the sea-type ecotype was 0.61 (observed 0.62), which was marginally lower than that of the lake-type ecotype (0.68, observed 0.67) and the Kokanee ecotype (0.68, observed 0.67).

**Table 1:** Mean number of alleles observed per locus at 14 microsatellite loci for sea-type Sockeye Salmon, lake-type Sockeye Salmon, and Kokanee standardized to a sample size of 275 per ecotype.

	Sea-type sockeye	Lake-type sockeye	Kokanee
*Ots107*	7.77	7.01	7.77
*Ots108*	19.00	21.83	20.41
*Ots100*	18.73	25.17	26.91
*Oki10*	29.00	27.76	30.89
*Oki16*	12.62	23.04	27.15
*Oki1a*	3.73	6.00	5.93
*Oki1b*	4.97	4.88	4.67
*Oki29*	22.95	28.07	29.04
*Oki6*	15.94	18.24	21.18
*Omy77*	11.97	14.67	12.82
*One8*	10.87	12.70	15.80
*Ots103*	19.84	21.80	24.81
*Ots2*	14.98	15.85	16.62
*Ots3*	10.51	13.68	15.59
Total	202.88	240.70	259.59

### Distribution of genetic variance

Gene diversity analysis of the 14 microsatellites surveyed was used to evaluate the distribution of genetic variation partitioned between the lake-type Sockeye Salmon and Kokanee ecotypes, among regions within ecotypes (10 regions Sockeye Salmon, 7 regions Kokanee, Table 2), among populations within regions (77 Sockeye Salmon populations, 42 Kokanee populations), and within populations. The amount of variation within populations ranged from 80.5% (*Ots100*) to 96.4% (*Oki10*), averaging 89.7%. Variation between the two ecotypes accounted for 2.04% of observed variation, which was not significant (P>0.05). However, variation among regions within ecotypes accounted for 5.36% of observed variation (P<0.01), and was the largest source of variation after within-population variation (Table 3). Differentiation between the two ecotypes was only 38% (2.04/5.36) of the magnitude of variation among regions within ecotypes. Variation among populations within regions was the next largest source of variation, and accounted for 2.93% of total observed variation. For populations in the Fraser River and Columbia River drainages, regional differences contributed more to differentiation of allele frequencies than ecotypes (lake-type Sockeye Salmon and Kokanee) or population sources of variation.

**Table 2:** River drainage, geographic region within drainage, population within region, sample collection years, and total number of fish sampled for 42 Kokanee populations (4,054 individuals), 77 lake-type Sockeye Salmon populations (22,048 individuals), and two sea-type Sockeye Salmon populations (411 individuals) in the Columbia River and Fraser River drainages.

Drainage	Region	Population	Years	N
Kokanee				
Columbia	Okanagan River	1) Mission Creek	1997 2003 2004 2005	263
		2) Powers Creek	2003 2004	97
		3) Peachland Creek	2004 2004	110
		4) Shingle Creek	2004 2005	68
		5) Equesis Creek	2004	41
		6) Deep Creek	2004	41
		7) Whiteman Creek	2004	45
		8) Nashwito Creek	2004	46
		9) Fintry Beach	2004	53
		10) Paul’s Tomb Beach	2004	84
		11) Rattlesnake Island	2003 2004	187
		12) Whiskey Island	2004	48
		13) Bertram Park	2003	101
		14) Okanagan River	2004 2005 2007 2012	488
		15) Skaha Lake	1999 2003 2004 2011 2012	281
	Kootenay River	16) Kikomun Creek	2004	100
		17) Norbury Creek	2003 2004	200
		18) Lussier Creek	2003	100
		19) Meadow Creek	2003 2004	297
		20) Kokanee Creek	2010	99
		21) Redfish Creek	2010	100
		22) Hill Creek	2010	61
Fraser	Nechako	23) Burns Lake	2000	66
		24) Fraser Lake	2000	18
		25) Endako River	1999 2000	76
		26) Stellako River	2000	30
	Quesnel Lake	27) Horsefly River	1992 2005	121
		28) Quesnel River	2005	13
		29) Deception Point	2005	39
	Mid Fraser	30) Elkin Creek	2010	17
		31) Anderson Lake	2003 2004	36
		32) Seton Lake	2003	40
	Thompson River	33) Mabel Lake	2004	63
		34) Mara Lake	2004	51
		35) Middle Shuswap Lake	2003	20
		36) Shuswap Lake	2004	98
		37) Adams Lake	2003	28
	Lower Fraser	38) Cultus Lake	2007 2008	38
		39) Chilliwack Lake	2004	100
		40) Alouette River	2000 2002 2007	104
		41) Coquitlam River	2004 2005	60
		42) Stave Lake	2009 2011	126
Lake-type Sockeye Salmon				
Columbia	Columbia	43) Osoyoos Lake	1993 1997 1998 1999 2000 2001 2002 2003 2004 2012	1068
		44) Redfish Lake	2008 2009 2010	200
		45) Bedrock Creek	1996	99
		46) Lake Wenatchee	1988 2007	89
		47) Rocky Reach	2005	80
Fraser	Early Stuart	48) Felix Creek	2005	99
		49) Paula Creek	2005	116
		50) Rossette Creek	2005	100
		51) Sandpoint Creek	2005	97
		52) Hudson Bay Creek	2000 2005	120
		53) Porter Creek	2000 2005	120
		54) Blackwater Creek	2000 2005	123
		55) Sinta Creek	2005	97
		56) Gluskie Creek	1997	151
		57) Five Mile Creek	2005	99
		58) Forfar Creek	1997	151
		59) Bivouac Creek	2005	99
		60) Driftwood Creek	2005	98
		61) Narrows Creek	2005	98
		62) Kynock Creek	1994 1997	180
		63) Dust Creek	1988 1991 1994 1997 2005	349
	Late Stuart/Stellako	64) Kuzkwa River	2001	104
		65) Tachie River	1994 1995 1996 1997 1999 2000 2001 2011 2012	682
		66) Pinchi Creek	1995 2005	171
		67) Stellako River	1992 1995 1996 1998 1999 2000 2011	689
		68) Middle River	1993 1996 1997 1998 2000 2001	425
		69) Nadina River	1986 1992 1999 2000	353
		70) Ormonde Creek	2010	24
	Quesnel	71) McKinley Creek	2001 2005	225
		72) Lower Horsefly River	2001	200
		73) Middle Horsefly River	2001	198
		74) Upper Horsefly River	2000 2001	497
		75) Horsefly River (mixed)	1985 1986 1993 1996 1997 1998 1999 2005	946
		76) Mitchell River	1993 1994 1996 1997 1998 2001 2005	537
		77) Blue Lead Creek	2001	100
		78) Roaring River	2001	100
		79) Wasko Creek	2001	100
		80) Deception Point	2005	77
	Chilko Lake	81) Chilko River north	1992 1995 1996 1997 1998 1999 2000 2001 2008 2009	1004
		82) Chilko River south	1996 1997 2001	410
		83) Taseko Lake	2007 2010 2011	126
	Mid/upper Fraser	84) Yohetta Creek	2010 2011	25
		85) Nemian Creek	2010	20
		86) Bowron River	1999 2000 2001	264
		87) Bridge River	2011	17
		88) Portage Creek	1986 1997 1998 1999	466
		89) Nathatlatch Lake	1996 1997 2010	338
		90) Nathatlatch River	2010	102
		91) Gates Creek	1986 1992 1995 1999 2000	433
	South Thompson	92) Upper Adams River	1996 2000 2010	466
		93) Lower Adams River	1982 1990 1995 1996 1998 1999	550
		94) Little River	2002	101
		95) Eagle River early	2000 2002	198
		96) Eagle River late	1986 1990 2002 2010	384
		97) Lower Shuswap River	1983 1986 1990 1996 1998 1999 2002	408
		98) Middle Shuswap River	1986 2002	246
		99) Salmon River	2010	30
		100) Seymour River	1986 1996 1999	335
		101) Anstey River	2010	98
		102) Scotch Creek	1994 1995 1996 1999 2000	536
		103) Sinmax Creek	2010	54
		104) Cayenne Creek	2000	100
	North Thompson	105) Raft River	1996 2000 2001 2012	319
		106) North Thompson River	2003 2005 2012	225
		107) Upper Barriere River	1996 1999 2000 2001	491
	Chilliwack River	108) Cultus Lake	1992 1995 1999 2000 2001 2002 2004 2005 2006 2007 2008 2009	2407
		109) Chilliwack Lake	1996 2003 2004 2005	226
		110) Dolly Varden Creek	2001 2003	121
	Harrison River	111) Birkenhead River	1992 1995 1997 1998 1999 2001 2010	644
		112) Weaver Creek	1982 1986 1992 1996 1998 1999 2000 2001	692
		113) Green River	2011 2012	95
		114) Sampson Slough	2010 2011 2012	163
		115) Cogburn Creek	2003 2011	29
		116) Big Silver Creek	2000 2002	199
		117) Douglas Creek	2002 2003 2011	19
	Pitt River	118) Pitt River	1986 2000 2001 2005 2010	447
		119) Corbold Creek	2010 2011	199
Sea-type Sockeye Salmon				
Fraser River	Lower Fraser	120) Harrison River	1985 1995 2000	329
		121) Widgeon Slough	2002	82

**Table 3:** Hierarchical gene-diversity analysis of 77 populations of lake-type Sockeye Salmon and 42 populations of Kokanee within 17 regions in the Columbia River and Fraser River drainages for 14 microsatellite loci.

Locus	Within	Among populations	Among regions	Between
	Populations	within regions	within ecotypes	ecotypes
*Ots107*	0.8263	0.0187**	0.0472**	0.1079*
*Ots100*	0.8053	0.0292**	0.0666**	0.0989*
*Ots3*	0.8834	0.0307**	0.0377**	0.0482*
*Oki1b*	0.8989	0.0256**	0.0343**	0.0412*
*Oki1a*	0.9017	0.0337**	0.0399**	0.0247
*Omy77*	0.8901	0.0342**	0.0654**	0.0103
*Ots2*	0.8999	0.0252**	0.0683**	0.0066
*Oki16*	0.8373	0.0379**	0.1214**	0.0035
*Oki10*	0.9685	0.0189**	0.0101**	0.0025
*Oki29*	0.9160	0.0329**	0.0511**	0.0000
*Oki6*	0.8642	0.0392**	0.0965**	0.0000
*One8*	0.9268	0.0255**	0.0477**	0.0000
*Ots103*	0.9491	0.0244**	0.0265**	0.0000
*Ots108*	0.9253	0.0326**	0.0421**	0.0000
*Total*	0.8967	0.0293**	0.0536**	0.0204

*P<0.05

**P<0.01

### Population structure

Substantial allelic frequency differentiation was observed among all three ecotypes of *O*. *nerka* examined, with the largest average *F*_*ST*_ value observed between the sea-type Sockeye Salmon and Kokanee ecotypes (*F*_*ST*_ = 0.170), next between the sea-type and lake-type Sockeye Salmon ecotypes (*F*_*ST*_ = 0.140), and finally between the lake-type Sockeye Salmon and Kokanee ecotypes (*F*_*ST*_ = 0.115) (Table 4). *F*_*ST*_ values per locus over all 121 populations were: Oki10 0.031, Ots103 0.049, Oki1b 0.072, One8 o.074, Ots108 0.075, Oki1a 0.080, Oki29 0.083, Ots3 0.088, Ots2 0.098, Omy77 0.107, Ots107 0.104, Ots100 0.135, Oki6 0.141, and Oki16 0.172, with an overall FST value of 0.087. Higher allelic frequency differentiation was observed among regional groups of Kokanee populations (average *F*_*ST*_ = 0.132) compared with regional groups of lake-type Sockeye Salmon populations (average *F*_*ST*_ = 0.087). The largest differentiation among populations within an ecotype and region was observed between the sea-type Sockeye Salmon Harrison River and Widgeon Slough populations (*F*_*ST*_ = 0.176). Populations within ecotypes and geographically similar locations (the diagonal of Table 4) displayed less differentiation than ecotype and regional comparisons, with generally significant genetic differentiation among regional stock comparisons within ecotypes.

**Table 4:** Mean pairwise *F*_*ST*_ values averaged over 14 microsatellite loci from 16 regional groups of Sockeye Salmon and Kokanee (*Oncorhynchus nerka*) that were sampled at 121 locations in the Fraser River and Columbia River drainages.

	1	2	3	4	5	6	7	8	9	10	11	12	13	14	15	16
1	**0.009**	0.039	0.072	0.043	0.038	0.051	0.097	0.118	0.090	0.089	0.074	0.101	0.086	0.100	0.095	0.125
2	0.017	**0.029**	0.086	0.052	0.037	0.060	0.109	0.127	0.123	0.114	0.078	0.116	0.106	0.109	0.112	0.134
3	0.034	0.038	**0.081**	0.084	0.084	0.088	0.118	0.153	0.140	0.126	0.110	0.131	0.122	0.137	0.129	0.152
4	0.017	0.023	0.038	**0.028**	0.046	0.059	0.106	0.106	0.113	0.096	0.067	0.102	0.083	0.100	0.094	0.133
5	0.005	0.014	0.040	0.018	**0.016**	0.058	0.096	0.122	0.121	0.092	0.079	0.110	0.103	0.108	0.105	0.133
6	0.006	0.014	0.039	0.020	0.008	**0.035**	0.104	0.117	0.124	0.116	0.065	0.098	0.082	0.104	0.092	0.106
7	0.022	0.020	0.044	0.028	0.021	0.024	**0.066**	0.165	0.157	0.140	0.134	0.130	0.142	0.140	0.151	0.167
8	0.042	0.041	0.051	0.042	0.033	0.044	0.040	**0.091**	0.190	0.188	0.121	0.158	0.128	0.158	0.167	0.172
9	0.034	0.038	0.042	0.044	0.040	0.036	0.041	0.052	**0.061**	0.137	0.140	0.153	0.143	0.136	0.136	0.195
10	0.020	0.027	0.033	0.030	0.024	0.024	0.028	0.057	0.046	**0.081**	0.140	0.150	0.130	0.129	0.130	0.203
11	0.009	0.018	0.027	0.022	0.010	0.016	0.029	0.059	0.036	0.046	**0.047**	0.119	0.083	0.108	0.084	0.144
12	0.025	0.030	0.038	0.034	0.028	0.024	0.043	0.042	0.042	0.044	0.026	**0.135**	0.124	0.124	0.129	0.162
13	0.012	0.015	0.033	0.021	0.011	0.011	0.020	0.048	0.026	0.032	0.014	0.029	**0.012**	0.103	0.095	0.153
14	0.025	0.026	0.024	0.034	0.023	0.031	0.023	0.054	0.030	0.035	0.034	0.031	0.038	**0.039**	0.093	0.167
15	0.012	0.015	0.023	0.022	0.010	0.018	0.024	0.053	0.033	0.038	0.023	0.028	0.018	0.023	**0.053**	0.168
16	0.079	0.079	0.084	0.088	0.078	0.076	0.072	0.090	0.083	0.101	0.100	0.078	0.101	0.073	0.083	**0.176**

Comparisons were conducted between individual populations in each region. Values in bold on the diagonal are comparisons among populations within each region. *F*_*ST*_ values are listed above the diagonal, with standard deviations below the diagonal. RC is region code (Sockeye Salmon are lake-type unless otherwise indicated), and codes are as follows: 1) Stuart River Sockeye Salmon, 2) Chilko/Quesnel/Middle Fraser Sockeye Salmon, 3) Gates/Nahatlatch Sockeye Salmon, 4) South Thompson Sockeye Salmon, 5) North Thompson Sockeye Salmon, 6) Harrison/Pitt Sockeye Salmon, 7) Chilliwack/Cultus Sockeye Salmon, 8) Columbia Sockeye Salmon, 9) Nechako/Stuart Kokanee, 10) Quesnel Kokanee, 11) Mid-Fraser/Anderson/Seton Kokanee, 12) Lower Fraser Kokanee, 13) Okanagan Kokanee, 14) Kootenay Kokanee, 15) South Thompson Kokanee, 16) Fraser sea-type Sockeye Salmon.

**Fig 1:**
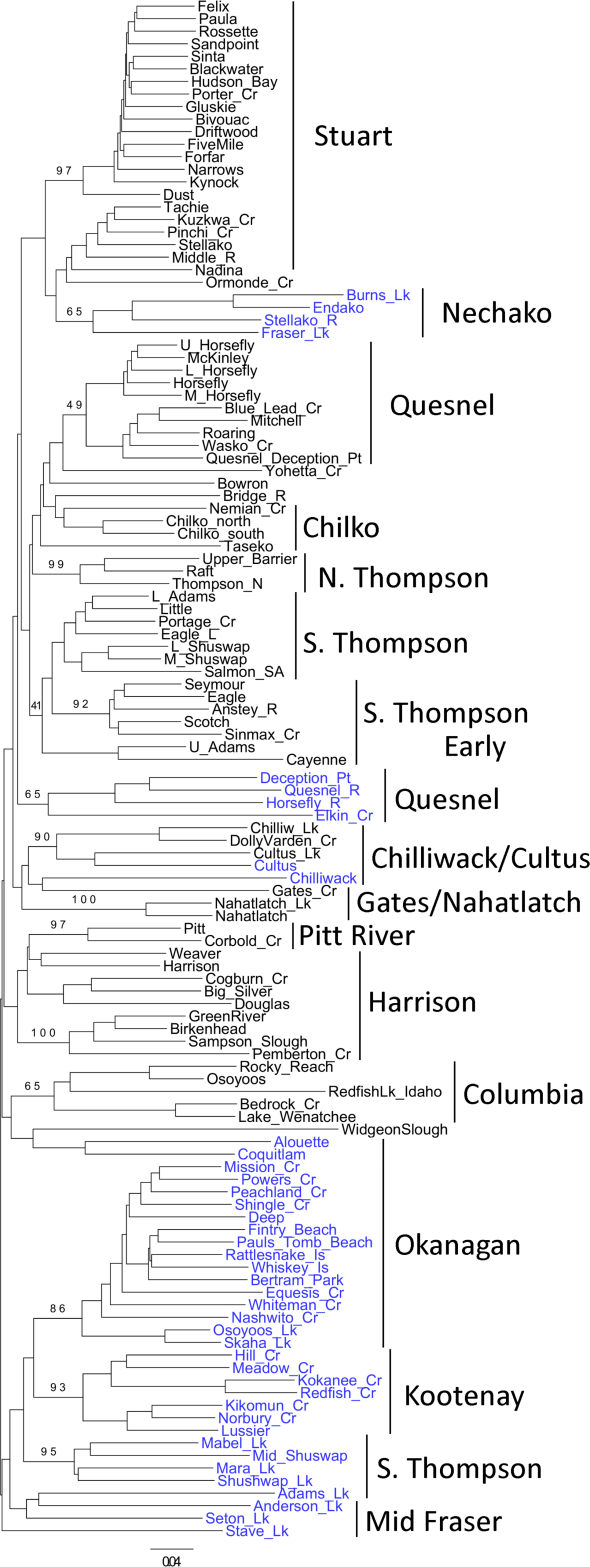
Neighbor-joining dendrogram of Cavalli-Sforza and Edwards (1967) chord distance for 80 populations of Sockeye Salmon and 42 populations of Kokanee from the Columbia River and Fraser River drainages surveyed at 14 microsatellite loci. Bootstrap values at major tree nodes indicate the percentages of 500 trees for which the populations beyond the node clustered together. Note the dendrogram proceeds vertically from one page to the next. Sockeye Salmon population names are in black, Kokanee population names are in blue. Harrison and Widgeon Slough are two sea-type Sockeye Salmon populations.

Population structure of the ecotypes was a function both of the ecotype and the region evaluated. For example, Kokanee populations from the Columbia River drainage (Okanagan Lake, Kootenay Lake), the South Thompson River drainage populations, and the mid-Fraser River populations all clustered together in the neighbor-joining tree (Fig 1). However, upstream of the mid-Fraser River populations, there were closer relationships between the lake-type Sockeye Salmon ecotype and the Kokanee ecotype, with the Nechako River Kokanee populations most similar to the Stuart River Sockeye Salmon populations. The sea-type Sockeye Salmon Harrison River population clustered with other lake-type Sockeye Salmon populations in the Harrison River drainage, and the sea-type Sockeye Salmon Widgeon Slough population in the lower Fraser River drainage was most similar to Kokanee populations in the lower Fraser River drainage.

## Discussion

With respect to the three main assumptions (1. sea-type ecotype ancestral with weakly differentiated populations; 2. genetically differentiated lake-type ecotype evolved repeatedly from the sea-type ecotype in parallel adaptive radiations; 3. Kokanee ecotype repeatedly evolved independently from the lake-type ecotype in a parallel adaptive radiation) of the evolution of O.nerka ecotypes outlined by [4], the results of our study can be summarized as follows. No evidence was found to support the hypothesis that the sea-type ecotype was comprised of weakly differentiated populations, nor was evidence available to suggest that the lake-type ecotype evolved repeatedly from the sea-type ecotype. Close genetic relationships between the lake-type ecotype and the Kokanee ecotype in the upper mid Fraser River drainage suggested that the lake-type ecotype could have been the ancestral form, but existing lake-type ecotypes may also have been derived from the Kokanee ecotype.

If the Kokanee ecotype is a result of parallel evolution, then pair-wise genetic distances between sympatric lake-type Sockeye Salmon and Kokanee should be less than among populations of the same ecotype in different lakes. However, since there can be contemporary gene flow between the ecotypes in some cases, it can be difficult to distinguish between parallel independent evolution of the two ecotypes versus a monophyletic origin of one ecotype with contemporary gene flow between ecotypes. Available genetic evidence indicates that there can be substantial differentiation between lake-type Sockeye Salmon and Kokanee within the same lake. Differentiation between the ecotypes in Takla Lake in the Fraser River drainage was substantially larger than variation among populations within ecotypes or among sampling years within populations [15]. Within one lake, the Kokanee ecotype was reported to be distinct from the lake-type ecotype, while in another lake, little differentiation was observed [16]. A survey of genetic variation between the lake-type and Kokanee ecotypes across a broad geographic range suggested parallel evolution between the lake-type and Kokanee ecotypes [17]. Significant genetic differentiation was observed between sympatric Sockeye Salmon and Kokanee in three separate localities [11]. The authors suggested that these two Kokanee populations may share a common monophyletic origin, relative to their sympatric Sockeye Salmon counterparts. They pointed out the close geographic proximity of the two river systems, < 20 km at some point, and suggested that stream capture may have occurred in recent geological time. They noted that, during the deglaciation of British Columbia, the Fraser and Columbia rivers were connected through a series of glacial lakes that formed in the Okanagan Valley [18]. Given the opportunities for gene exchange between these two river basins, gene flow may account for the genetic similarity between these Sockeye Salmon and Kokanee populations. In the current study, when one examines the population structure of the Kokanee ecotype as depicted in the dendrogram, the simplest explanation is that there was a monophyletic radiation of the ecotype in a portion of the Fraser River and Columbia River drainages, as shown by the clustering of populations in the Okanagan River, Kootenay River (Columbia River tributaries), and South Thompson River (Fraser River tributary). These results support those of [17], who suggested that Kokanee populations in these two river drainages may share a common monophyletic origin, relative to their sympatric Sockeye Salmon counterparts. However, when the entire Fraser River drainage is evaluated, the dendrogram suggests that there have been several independent evolutionary derivations of the Kokanee ecotype. Differentiation between the two ecotypes (2.04% of total observed variation) was only 38% of the magnitude of variation among regions within ecotypes (5.36% of total observed variation), suggestive of a polyphyletic origin of the ecotypes. Population structure within the lake-type ecotype is stable over time, as differentiation among river drainages and populations within river drainages has been reported to be approximately 19 times greater than that of annual variation within populations [13].

Has the Kokanee ecotype been derived repeatedly from the lake-type Sockeye Salmon ecotype? If so, then one may expect differences in genetic characters and possibly morphological characters between the ecotypes within a region to be less than differences among regions within an ecotype, particularly if there is contemporary gene flow between the ecotypes within a lake [19]. Differences in gill raker number between the lake-type and Kokanee ecotypes in Takla Lake in the Fraser River drainage have been examined [15, 20]. Both studies reported mean gill raker counts of 39.5–39.7 gillrakers for the Kokanee ecotype, and 36.2–36.5 gill rakers for the lake-type Sockeye Salmon ecotype, with the difference of three gill rakers the largest known to occur between sympatric ecotypes [10]. In a broad survey of variation in the number of gill rakers of Sockeye Salmon in North America, the mean number of gill rakers for Fraser River Sockeye Salmon was 36.5, with regional variation in the ecotype ranging from 34.4 (Adak Island) to 37.1 (Bristol Bay) [21]. Greater differences in gill raker number were reported between the sympatric ecotypes within Takla Lake than within the lake-type Sockeye Salmon ecotype over thousands of km in geographic distance. The distribution of gill raker phenotypes between the ecotypes did not support a finding of less differentiation between the ecotypes within a region than differences among regions within an ecotype. However, gill raker number may be subject to selection, and as such may not provide a reliable indicator on the plylogeny of the ecotypes.

There can be uncertainity in our study as to which ecotype was surveyed in a particular lake. For example, *O*. *nerka* from the Stave, Coquitlam, and Alouette lakes were defined as the Kokanee ecotype, largely because dams constructed in the watersheds blocked access for anadromous (lake-type) Sockeye Salmon to the lakes in the drainages. A dam downstream from Stave Lake was completed in 1912, with the Coquitlam Lake dam completed in 1914, and the Alouette Lake dam completed in 1927. The lake-type ecotype had been present in the watersheds prior to the construction of the dams, but once the dams were completed, access to the lakes was blocked, and the life cycle of *O*. *nerka* upstream from the dams was completed entirely in fresh water, hence the designation of the Kokanee ecotype in our study. However, experimental water releases past the dams on the Alouette and Coquitlam rivers in 2005 and 2006 resulted in juveniles passing the dams, which resulted in return migrations of anadromous adults in 2007 and 2008 after nearly 90 years of an entirely freshwater life cycle [22]. These three populations were among the most genetically atypical of either the lake-type Sockeye Salmon or Kokanee ecotypes surveyed (Fig 1). Designation of these populations as the lake-type Sockeye Salmon ecotype would not have altered the basic conclusions of the study.

Earlier surveys of allozyme variation in *O*. *nerka* indicated that there was little genetic differentiation in populations of the sea-type ecotype, even though populations were sampled across a broad geographic range of about 2,000 km [12]. If this were the consistent finding across studies, then indeed that this would suggest that there is a substantial amount of straying among populations of the sea-type ecotype, and this level of straying may lead to colonization of new habitats from which the lake-type and Kokanee ecotypes could evolve. However, lack of genetic differentiation among populations of the sea-type ecotype does not appear to be the general pattern of population structure in *O*. *nerka* [13, 23]. The sea-type ecotype is more common in northern rivers in glaciated regions where lake habitat is absent or non-productive [2]. In the Alsek River drainage in northern British Columbia, all sea/river type populations are distinct from all other populations in northern British Columbia [14], which does not support the concept of limited differentiation of populations of the sea-type ecotype across a broad geographic range. Furthermore, there was observed genetic differentiation among sea/river ecotype populations across British Columbia [13, 14], such that there is little support for a notion of limited differentiation among populations of this ecotype over a wide geographic area. In the current study, the pairwise *F*_*ST*_ value between the Harrison Rapids (River) and Widgeon Slough populations, both populations located in the lower Fraser River drainage, was 0.176, among the largest observed in the study. In British Columbia, population structure of *O*. *nerka* sea/river ecotype populations does not appear to support the notion of one large metapopulation, with relatively genetically undifferentiated populations.

Within a drainage, there are sometimes, but not always, genetic similarities between sea/river populations and lake-type populations. This association has been demonstrated for populations within the Alsek River drainage in northern British Columbia, where all 16 populations surveyed, including both ecotypes, clustered together in a dendrogram analysis of population structure [13]. This was also evident in the current study, where the sea/river type Harrison Rapids population clustered with all other lake-type populations in the Harrison River drainage. Within a drainage, there may be cases where a sea/river type population, located lower in the river drainage, is genetically similar to a dominant lake-type population higher in the drainage. This exact situation occurs in the Skeena River drainage in northern British Columbia. The Halliday Slough population, located below Babine Lake in the Skeena River and to which the population has no lake access, is similar genetically to the dominant Babine Lake population (unpublished data), which comprises 85% of Skeena River drainage escapement [24]. The 10 populations sampled in the Babine Lake complex are quite distinct genetically from the other 17 populations sampled in the drainage[24], but the Halliday Slough population is the only population ever surveyed in the drainage which displayed genetic similarity to the complex of Babine Lake populations. As there are no known fry migrants from the lake, it seems plausible that this population was initially founded from the lake-type ecotype that was migrating upstream to Babine Lake but simply ran out of energy reserves to complete migration, spawning in the available habitat in Halliday Slough, giving rise to the small present-day population. In this example, the sea-type ecotype was not the ancestral form, and lake-type Sockeye Salmon in Babine Lake have not evolved from the sea-type or river-type ecotype in parallel adaptive radiations. Rather, the reverse situation likely occurred, with the sea-type or river-type ecotype arising from the lake-type ecotype.

Kokanee populations in the Columbia River and South Thompson River may share a common monophyletic origin. With respect to the entire Fraser River drainage, if one excludes Kokanee populations in the lower Fraser River drainage, as these were lake-type ecotypes turned Kokanee ecotypes by dam construction [22] or otherwise altered by transplantation, and if one assumes a monophyletic origin of the Kokanee ecotype surveyed in the current study in the Columbia and South Thompson rivers, then the question arises as to why there are genetic similarities between lake-type Sockeye Salmon and Kokanee ecotypes in the middle portion of the Fraser River drainage (Stuart River lake-type and Nechako River Kokanee; Quesnel Lake lake-type and Kokanee) as noted in the current study. Geologic evidence from sites upstream of Texas Creek in the middle Fraser River drainage suggested that the Fraser River was dammed by avalanche debris and that one of these events occurred about 1200 yrs ago [25]. This may have prevented the upstream migration of salmon, since widespread collapse of First Nation culture occurred here at about that time [25]. If so, then the anadromous lake-type ecotype would have likely been exterminated from all lakes upstream of the postulated Texas Creek slide, and these lakes would have remained inaccessible until the presumed catastrophic destruction of the dam. It is possible that the closer relationship between lake-type Sockeye Salmon and Kokanee in the middle portion of the Fraser River drainage was a result of the existing Kokanee ecotype giving rise to the current lake-type Sockeye Salmon in this portion of the drainage, similar to the Kokanee ecotype in the dammed lakes in the lower portion of the Fraser River drainage giving rise to the newly developed lake-type anadromous Sockeye Salmon ecotype in the region.

In summary, the level of genetic differentiation observed among and within sea-type Sockeye Salmon, lake-type Sockeye Salmon, and Kokanee ecotypes suggested that there was little straying among populations within ecotypes, and limited introgression among ecotypes. Available evidence provides little support for the concept of the sea-type ecotype being the non-differentiated ancestral form of *O*. *nerka* owing to considerable genetic differentiation among populations within this ecotype. With ocean access blocked and later subsequently restored, the Kokanee ecotype can give rise to the lake-type Sockeye Salmon ecotype. Kokanee population structure within the Fraser River drainage suggested a polyphyletic origin of the ecotype within the drainage. The previous conclusions drawn from other studies about relationships among and within *O*. *nerka* ecotypes has been dependent upon the class of genetic variants employed (allozymes, minisatellies, and microsatellites) and the geographic scale of the survey of populations undertaken. Conclusions from studies employing older genetic technologies and restricted population sampling have not been confirmed by more recent surveys of variation within the ecotypes. Studies employing geographically restricted population sampling may not outline accurately the phylogenetic history of salmonid ecotypes, and larger-scale geographic sampling of populations is preferable in order to survey all variation that may be present within an ecotype.

## Methods

### Collection of DNA samples and laboratory analysis

**Fig 2:**
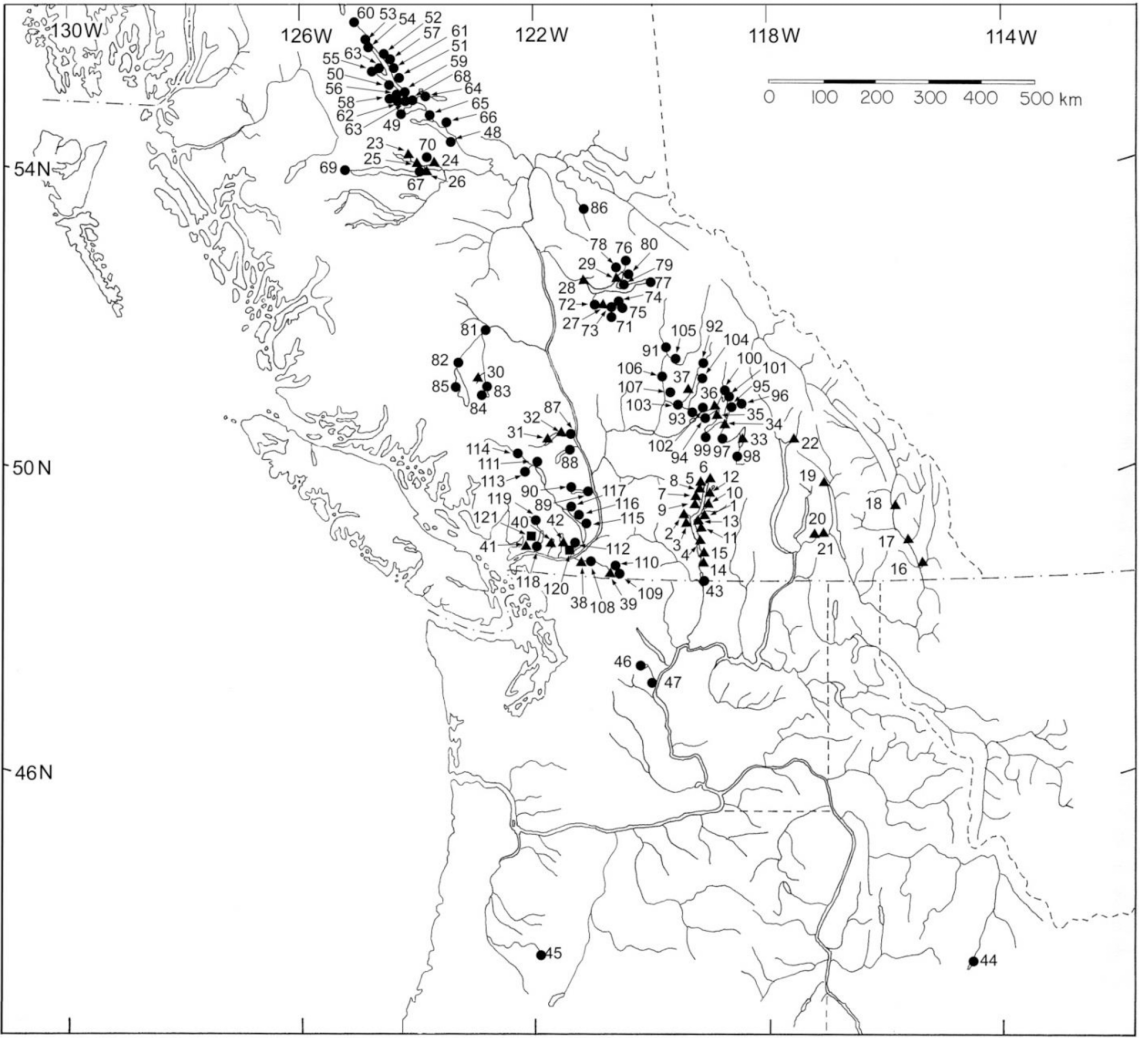
Map indicating sampling locations for 42 populations of Kokanee and 79 populations of Sockeye Salmon and in the Fraser River and Columbia River drainages. The specific populations in each drainage are outlined in Table 3. Triangles on map indicate resident Kokanee populations, circles are anadromous Sockeye Salmon populations.

Authorization to collect samples in the study was provided by a scientific license issued under the provisions of the Fisheries Act passed by the Canadian Parliament in 1985 and last amended in 2016. Under the Act, the scientific license was issued by Fisheries and Oceans Canada in order to allow Departmental staff to collect samples in the course of their work. As there is no requirement for an Institutional Animal Care and Use Committee (IACUC) or equivalent under the Act, sampling protocols were neither vetted nor approved by an IACUC. Sockeye Salmon and Kokanee are not an endangered or protected species in Canada. Fin clips or operculum punches were collected from recently dead or moribund adult fish on the spawning grounds in all populations surveyed in the study. Samples of Sockeye Salmon from the United States of America portion of the Columbia River drainage were provided by National Marine Fisheries Service (NMFS) staff of the National Oceanic and Atmospheric Administration following NMFS sampling protocols. DNA was extracted from the tissue samples as described by [26]. The study included a survey of microsatellite variation for over 26,000 fish from 121 populations in the Fraser River and Columbia River drainages (Fig 2). The specific populations, collection years, and sample sizes included in the survey are outlined in Table 3. PCR products at 14 microsatellite loci: *Ots2*, *Ots3* [27], *Ots100*, *Ots103*, *Ots107*, *and Ots108* [28, 29], *Oki1a*, *Oki1b*, Oki6, *Oki10*, *Oki16*, and *Oki29* [30, 31], *One8* [32], and *Omy77* [33] were size fractionated on denaturing polyacrylamide gels and allele sizes initially determined with the ABI 377 automated DNA sequencer. Allele sizes were determined with Genescan 3.1 and Genotyper 2.5 software (PE Biosystems, Foster City, CA). Later in the study, microsatellites were size fractionated in an ABI 3730 capillary DNA sequencer, and genotypes were scored by GeneMapper software 3.0 (Applied Biosystems, Foster City, CA) using an internal lane sizing standard. Allele identification between the two sequencers was standardized by analyzing approximately 600 individuals on both platforms and converting the sizing in the gel-based data set to match that obtained from the capillary-based set.

### Data analysis

All annual samples available for a location were combined to estimate population allele frequencies, as was recommended by [34], as variation among Sockeye Salmon populations within drainages and among drainages was 19 times greater than variation among sampling years within populations [12]. The genotypic frequencies at each locus generally conformed to those expected under Hardy-Weinberg equilibrium [12]. *F*_*ST*_ estimates [35] for each locus over all populations were calculated with FSTAT version 2.9.3.2 [36]. The significance of the multilocus F_ST_ value over all samples was determined by jackknifing over loci. Cavalli-Sforza and Edwards chord distance (CSE) [37] was used to estimate genetic distances among all populations. An unrooted neighbor-joining tree based upon CSE was generated using NJPLOT [38]. Bootstrap support for the major nodes in the tree was evaluated with the CONSENSE program from PHYLIP based upon 500 replicate trees [39]. FSTAT was used to measure the ‘allelic richness’ (allelic diversity standardized to a sample size of 275 fish, all populations sampled within each ecotype combined) for each ecotype evaluated. Testing of significance was conducted by excluding populations with fewer than 20 individuals sampled, keeping populations separate within ecotypes, standardizing to a sample size of 27 fish, and employing a variance ratio (F-test) to test for differences among ecotypes. Computation of the number of alleles observed per locus was carried out with FSTAT. The distribution of genetic variation in lake-type Sockeye Salmon and Kokanee ecotypes was evaluated between ecotypes, among regions within ecotypes, and among populations within regions. Estimation of variance components of ecotype differentiation, among regions within ecotypes, and among populations within regions was determined with GDA [40]. A variance ratio was used to test significance of the different hierarchical levels. Allele frequencies for all populations surveyed in the study are available via DRYAD doi identified as: data package title: Data from: Population structure of sea-type and lake-type Sockeye Salmon and Kokanee in the Fraser River and Columbia River drainages. Provisional DOI: doi:10.5061/dryad.3g824 Data files: Baseline Allele Frequencies.
